# Supplementary material for: Closed-Loop Microreactor on PCB for Ultra-Fast DNA Amplification: Design and Thermal Validation
Source: Micromachines (Basel). 2023 Jan 10;14(1):172. doi: 10.3390/mi14010172 (PMC9860919; doi:10.3390/mi14010172)
Supplement: Supplementary file 1 [file micromachines-14-00172-s001.zip › micromachines-2127880-supplementary.pdf]

Supplementing material for

Closed loop microreactor on PCB for ultra-fast DNA amplification: design and thermal validation

P. Skaltsounis [1,2], G. Kokkoris [1], T.G. Papaioannou [2], A. Tserepi [1]

[1] Institute of Nanoscience and Nanotechnology, National Center of Scientific Research (NCSR) “Demokritos”, Patr. Gregoriou E’ and 27 Neapoleos str., 15341 Aghia Paraskevi, Attiki, Greece

[2] School of Medicine, National and Kapodistrian University of Athens (NKUA),
75 Mikras Asias str., 11527 Athina, Attiki, Greece

S1. Mesh independency

To obtain mesh independency in the computational study, three free meshes (with tetrahedral elements) with different total number of elements were used for each microreactor geometry. These three meshes have approximately 1.5, 3, and over 5 million elements, with small deviations between the microreactor geometries. At the same time, for every microreactor geometry, three checking points were taken inside the microchannel, in order to check if their temperatures show significant changes by changing the number of mesh elements. The points were selected to be in a circle that passes through the center of the microchannel, and between the thermal zones (one point for each zone transition). More specifically the points are located exactly at the places where the temperature changes become sharper during the computational study, providing thus the best temperature sensitivity. This way, any change in temperatures due to mesh elements numbers will be more easily noticed.

In Figures S1-S3, the diagrams of temperature versus the number of mesh elements are presented, for each microreactor geometry.

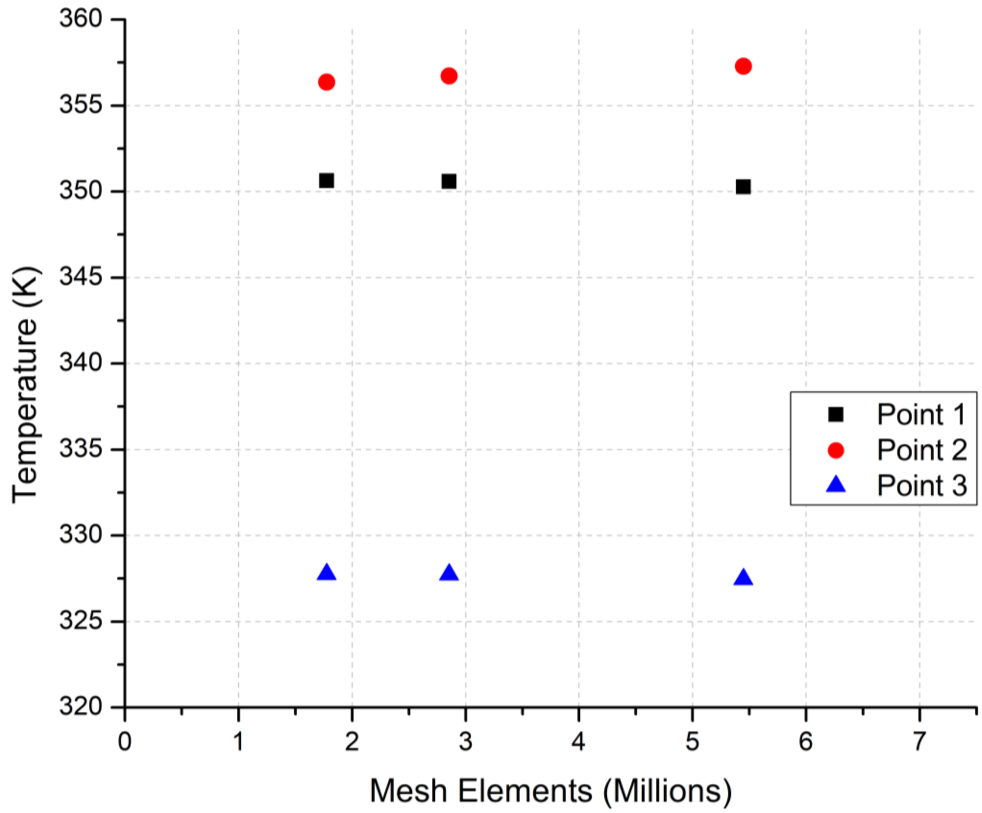

Figure S1. Temperature vs number of mesh elements: Microreactor 1.

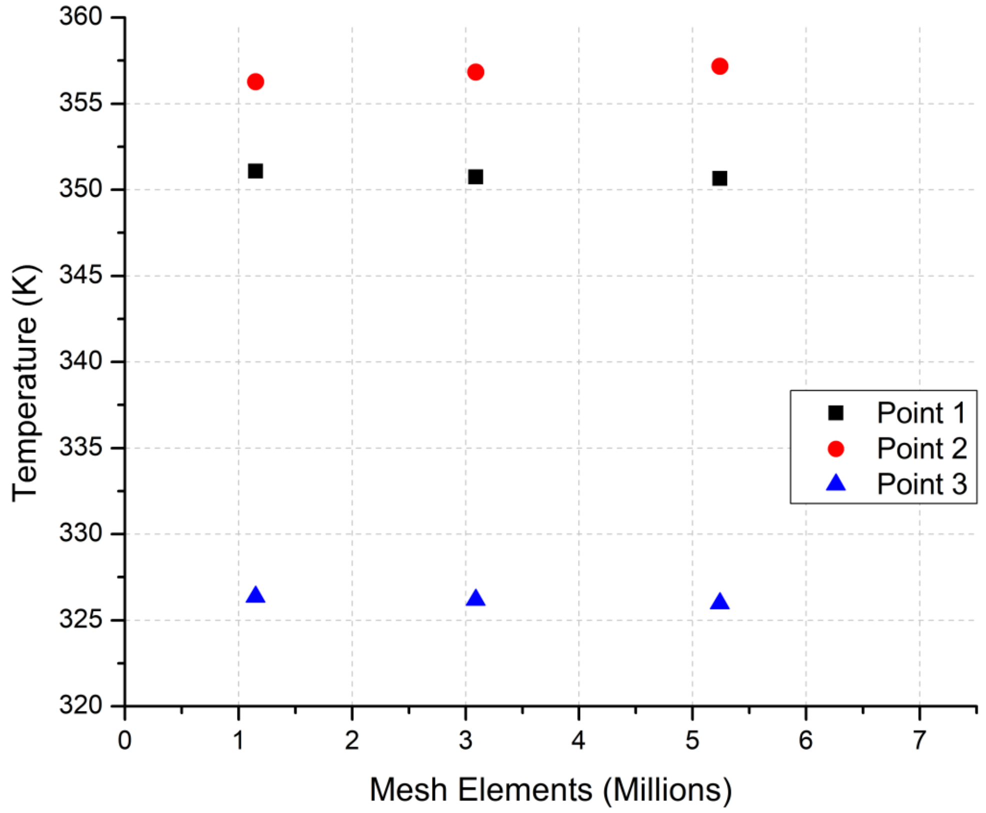

Figure S2. Temperature vs number of mesh elements: Microreactor 2.

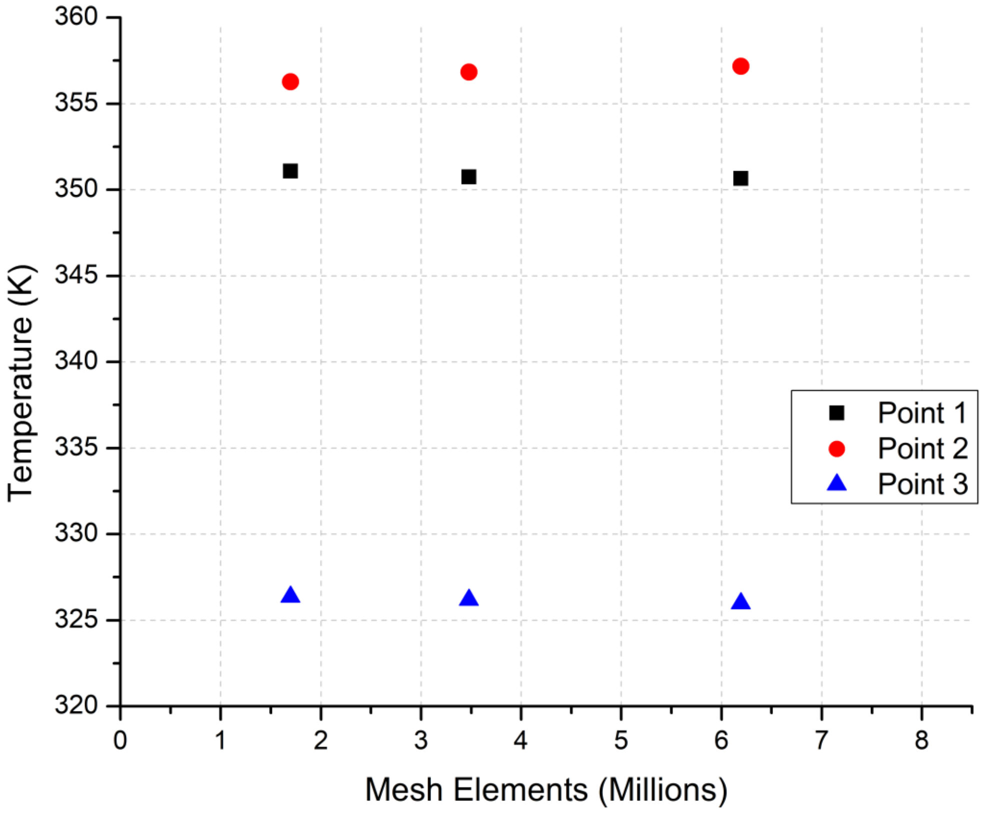

Figure S3. Temperature vs number of mesh elements: Microreactor 3.

It can be noticed that the temperature changes very little by increasing the number of mesh elements. In fact, the biggest temperature difference that occurs in all three diagrams is ΔT=0.57 K (microreactor 1, point 2, between meshes 1 and 2). This temperature difference is equivalent to 0.16 % and can be safely considered negligible for the results of this study. All the other temperature differences that have been examined are even smaller.

Apart from temperatures, total residence times were also examined to see if the results depend on the number of elements. Figure S4 shows in a diagram the values of total residence times that were obtained for different number of mesh elements, for all three microreactor geometries.

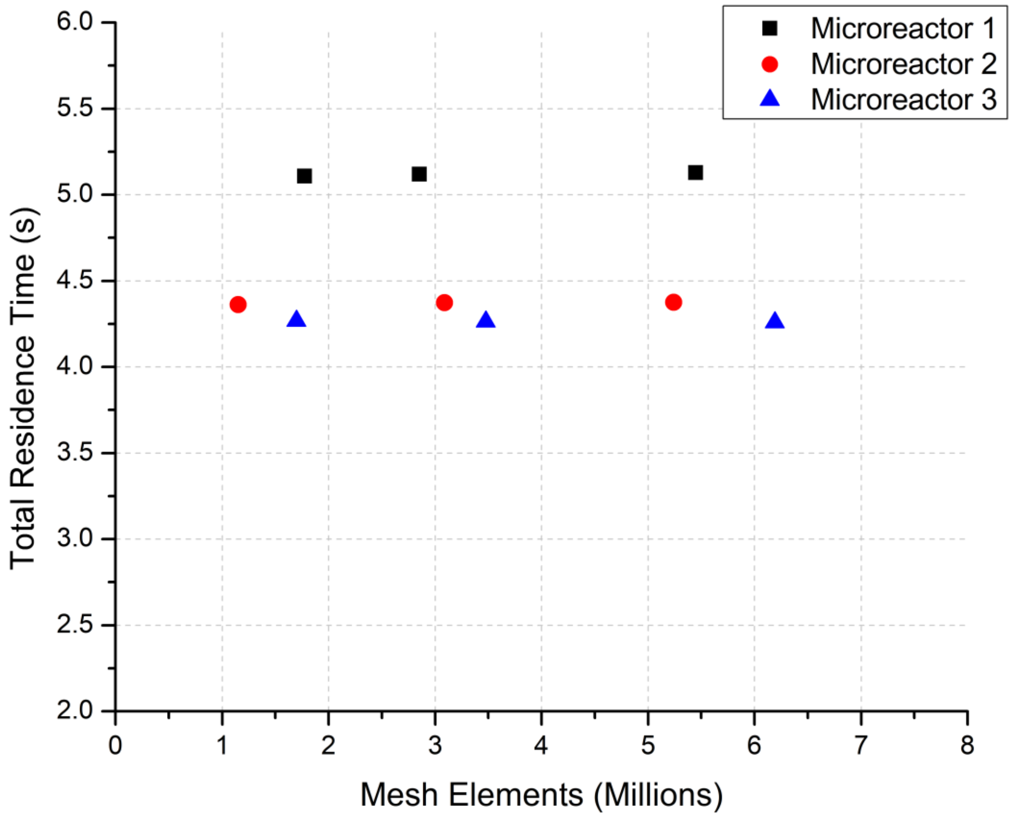

Figure S4. Total residence time vs number of mesh elements.

The differences in the total residence times between the first two meshes (approximately 1.5 million to 3 million elements) are in percentage: 1.15%, 1.14%, and 0.43% for microreactors 1, 2, and 3 respectively. These differences are reduced to 0.91%, 0.12%, and 0.41% between the last two meshes (approximately 3 million to over 5 million elements).

Once again, the differences that occur in the results can be considered negligible for the aim of this study.

Taking into consideration the above diagrams, we can safely conclude that the computational study provides mesh independent results, already from the less dense meshes (approximately 1.5 million elements). Nonetheless, the results presented in the main study, are obtained with the use of the denser meshes (over 5 million elements) to provide even higher accuracy, as computational resources were not a limiting factor for conducting this study.

S2. Heat Generation in microheaters

For all of the examined cycle times, the heat generation rates by the two microheaters were adjusted, by means of an iterative procedure, so that maximum residence times would be achieved for the denaturation and annealing steps. The following table shows the calculated heat generation rates of each heater and each microreactor design for the different cycle times.

Table S1. Heat generation rates in each microheater for different cycle times

Cycle time (s)	Power produced by the microheater (W)	
	Denaturation microheater	Extension microheater
	**Microreactor 1**	
6.9	0.881	0.588
7.0	0.879	0.587
7.1	0.877	0.587
7.2	0.875	0.587
7.3	0.874	0.587
7.4	0.873	0.586
7.5	0.871	0.586
	**Microreactor 2**	
6.6	0.735	0.646
6.7	0.733	0.646
6.8	0.731	0.646
6.9	0.729	0.646
7.0	0.727	0.645
7.1	0.725	0.646
7.2	0.724	0.644
	**Microreactor 3**	
5.1	0.788	0.652
5.2	0.785	0.651
5.3	0.782	0.650
5.4	0.779	0.649
5.5	0.776	0.648
5.6	0.773	0.647
5.7	0.771	0.646

S3. Geometry of the PCB chip without circular microchannel

Figure S5 shows the geometry of the fabricated PCB chip. This chip is covered with a very thin layer of solder mask and bears no circular microchannel on top of it. This geometry was used for the second computational study, which led to the comparison with the experimental results, and where no sample circulation took place.

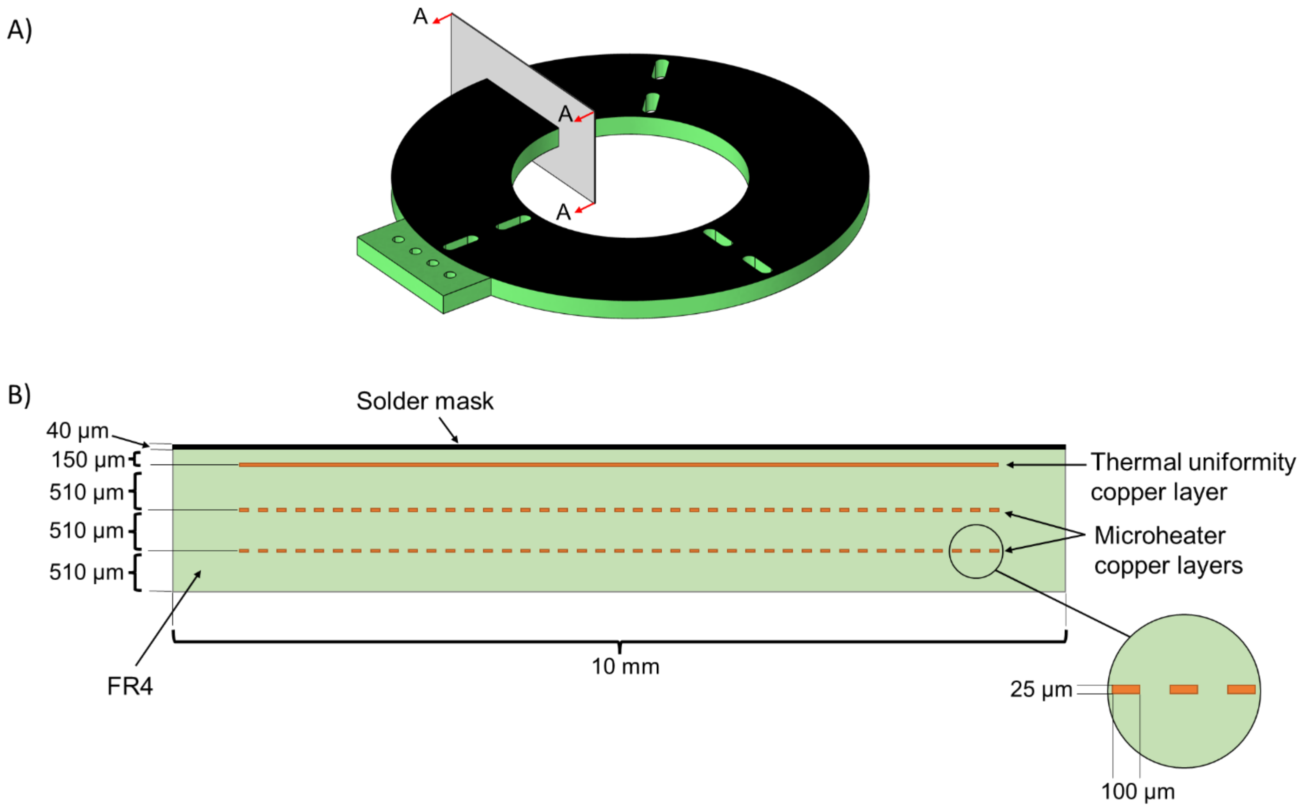

Figure S5. A) Geometry of the PCB chip, covered with the shoulder mask (without the circular microchannel). B) Cross section of the chip with the A-A plane.
